# Missed Opportunities for TB Investigation in Primary Care Clinics in South Africa: Experience from the XTEND Trial

**DOI:** 10.1371/journal.pone.0138149

**Published:** 2015-09-18

**Authors:** Violet N. Chihota, Sibuse Ginindza, Kerrigan McCarthy, Alison D. Grant, Gavin Churchyard, Katherine Fielding

**Affiliations:** 1 The Aurum Institute, Johannesburg, South Africa; 2 School of Public Health, Faculty of Health Sciences, University of the Witwatersrand, Johannesburg, South Africa; 3 London School of Hygiene & Tropical Medicine, London, United Kingdom; Institute of Infectious Disease and Molecular Medicine, SOUTH AFRICA

## Abstract

**Setting:**

40 primary health clinics (PHCs) in four provinces in South Africa, June 2012 –February 2013.

**Objective:**

To determine whether health care worker (HCW) practice in investigating people with TB symptoms was altered when the initial test for TB was changed from smear microscopy to Xpert MTB/RIF.

**Design:**

Cross-sectional substudy at clinics participating in a pragmatic cluster randomised trial, Xpert for TB: Evaluating a New Diagnostic "XTEND", which evaluated the effect of Xpert MTB/RIF implementation in South Africa.

**Methods:**

Consecutive adults exiting PHCs reporting at least one TB symptom (defined as any of cough, weight loss, night sweats and fever) were enrolled. The main outcome was the proportion who self-reported having sputum requested by HCW during the clinic encounter just completed.

**Results:**

3604 adults exiting PHCs (1676 in Xpert arm, 1928 in microscopy arm) were enrolled (median age 38 years, 71.4% female, 38.8% reported being HIV-positive, 70% reported cough). For 1267 participants (35.2%) the main reason for attending the clinic was TB symptom(s).

Overall 2130/3604 (59.1%) said they reported their symptom(s) to HCW. 22.7% (818/3604) reported having been asked to give sputum for TB investigation. Though participants in the Xpert vs. microscopy arm were more likely to have sputum requested by HCW, this was not significantly different: overall (26.0% [436/1676] vs 19.8% [382/1928]; adjusted prevalence ratio [aPR] 1.31, [95% CI 0.78–2.20]) and when restricted to those presenting at clinics due to symptoms (49.1% [260/530] vs 29.9% [220/737]; aPR 1.38 [0.89–2.13]) and those reporting being HIV-positive (29.4% [190/647] vs 20.8% [156/749]; aPR 1.38[0.88–2.16]).

Those attending clinic due to TB symptoms, were more likely to have sputum requested if they had increasing number of symptoms; longer duration of cough, unintentional weight loss and night sweats and if they reported symptoms to HCW.

**Conclusions:**

A large proportion of people exiting PHCs reporting TB symptoms did not get tested. Implementation of Xpert MTB/RIF did not substantially change the probability of testing for TB. Better systems are needed to ensure that opportunities to identify active TB among PHC attendees are not missed.

## Background

The burden of tuberculosis (TB) remains high in South Africa, with an estimated prevalence of 715 per 100 000 population in 2013 [1]. Despite better TB diagnostics, many people with TB remain undiagnosed or diagnosed but not started on appropriate treatment [1]. Control of TB in these high prevalence settings depends on early diagnosis and prompt initiation of appropriate treatment.

To reduce the burden of TB the World Health Organization (WHO) recommends intensified case finding (ICF) for TB among human immunodeficiency virus (HIV) infected persons [2, 3] and has also been recommended for all clinic attendees, regardless of HIV status [4]. Screening for TB is also an important step in triaging potentially infectious individuals at health facilities as part of infection control [5].

The Xpert MTB/RIF assay (Cepheid, Sunnyvale, CA), an automated nucleic acid amplification test that can detect both *Mycobacterium tuberculosis* [MTB] and rifampicin resistance, has very good sensitivity and specificity [6, 7] and can contribute to improving case finding. Since 2011, South Africa has rolled out Xpert MTB/RIF as the first-line sputum test for diagnosis of TB across the country, replacing smear microscopy. Embedded within this roll out was the XTEND trial, an evaluation of the effect of Xpert MTB/RIF implementation on patient-relevant outcomes [8]. The trial was a pragmatic, two arm, parallel, cluster-randomised trial where a cluster was defined as a laboratory and two clinics served by, but not co-located with, the laboratory. The primary outcome, mortality, was determined at six months among clinic attendees being investigated for TB.

Given that the outcomes of testing with Xpert MTB/RIF are critically dependent on the characteristics of the population having sputum sent for investigation, we needed to understand whether the process of implementing Xpert MTB/RIF changed health care worker (HCW) behaviour in requesting sputum for laboratory investigation. We reasoned that introduction of a new TB diagnostic test widely publicised as more sensitive could potentially encourage HCW to test more people for TB. Alternatively, knowledge that the new test was more expensive could influence HCW to ration its use. Therefore within the XTEND study, in this exit interview substudy, we aimed to determine, among adults exiting primary health clinics (PHCs) who reported at least one TB symptom, i) the proportion having sputum requested by an HCW by study arm and ii) factors associated with having sputum requested.

## Methods

### Ethics Statement

This study was approved by the ethics committees of the University of the Witwatersrand; the University of Cape Town, the London School of Hygiene and Tropical Medicine; and the World Health Organization. All consenting participants gave written consent or, for illiterate participants, witnessed verbal consent. For illiterate participants, there was an impartial witness present during the consenting process, who then signed the relevant witness section of the consent form. Both ethics committees approved the consent form, including the section on the use of witnessed oral consent for illiterate participants, at the beginning of the study. Principles expressed in the Declaration of Helsinki were followed in the conduct of this research.

### Study Design: The XTEND Study

In the XTEND study, a cluster was defined as a laboratory and two (PHCs) served by that laboratory. Laboratories were identified from four provinces, Gauteng, Mpumalanga, Free State and Eastern Cape provinces, and randomised to Xpert or microscopy as previously described [8].

### Study Design: Exit Interview Substudy

This was a cross-sectional study among adults exiting clinics at all 40 PHCs in both Xpert and microscopy arms.

### Study Population

Consecutive adults (≥18 years) leaving PHCs were screened for the exit interview substudy. A consecutive sample of those who reported to the research team at least one symptom suggestive of TB predefined according to WHO criteria, (i.e. cough≥24 hours, night sweats, weight loss and fever, hereafter referred to as “TB symptoms”) [2] were invited to take part in the study and enrolled if they were attending the clinic for personal health issues rather than accompanying a relative or friend. We excluded participants who had had sputum sent for TB investigations prior to the current visit or were already on TB treatment.

### Study Procedures

Data on demographics, additional information on TB symptoms and other clinical data relevant to TB were collected through an interview. Participants were also asked if they had had sputum requested by a HCW at that visit, and if sputum had not been requested, they were referred back to the clinic staff for appropriate investigations.

### Study Outcomes

The main outcome was the proportion of participants who had sputum requested by a HCW. Sensitivity analyses were conducted among three subgroups: those who reported that (i) their main reason for attending the clinic was because they had at least one TB symptom (ii) they had reported a TB symptom to HCW; iii) self-reported cough and reported cough to HCW (iv) they were HIV-positive.

### Data Management

All data including participant identifiers and enrolment questionnaire were collected using a custom designed data collection application on a smart phone as previously described [8]. Completed forms were submitted in an encrypted format via the cell phone network into a central repository database maintained at a contracted software company.

### Statistical Considerations

We assumed 10 clusters per arm (as dictated by the sample size for the XTEND study). We estimated that among clinic attendees reporting TB symptoms in the microscopy arm, 25% would have sputum requested by a HCW. With 200 clinic attendees per cluster and a coefficient of variation of 0.25, we estimated there would be 90% power to detect an absolute reduction greater than 10% or increase of at least 15% in TB testing in the Xpert arm.

The statistical analysis was conducted using STATA (version 12, StataCorp LP, College Station, Texas). The effect of study arm on the outcome for the main and sensitivity analyses used methods appropriate to the trial design with a small number of clusters [9], including adjustment for individual level baseline factors showing imbalance by study arm.

### Analysis of Factors Associated with Having Sputum Requested by HCW

Among participants whose main reason for attending the clinic was TB symptoms, a risk factor analysis was conducted using logistic regression with random effects to identify individual level factors associated with having sputum requested by HCW. The random effects model took into account clustering of patients within clinics. Factors with evidence of an association with the primary outcome (p value <0.05) in the univariable analysis were included in the multivariable model and adjusted odd ratios (aOR) and 95% confidence intervals (CIs) estimated. *A priori*, sex and age group were included in the fully-adjusted model.

## Results

### Participants

**Fig 1:**
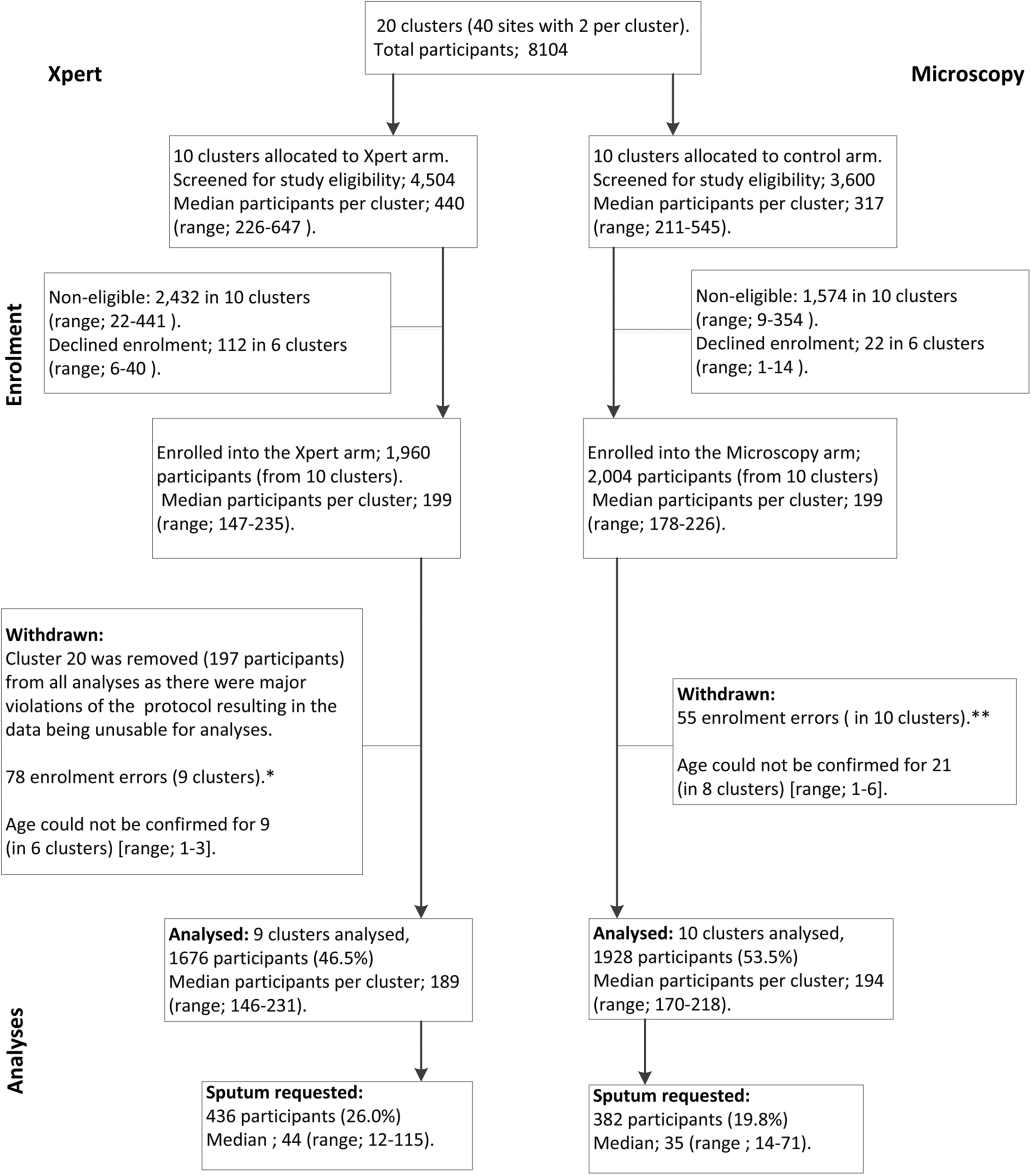
Participant flow for the exit interview substudy. Legend: *Participant enrolment errors, Xpert arm: did not complete survey n = 2; underage n = 10; attending clinic for TB care n = 66. **Participant enrolment errors, Microscopy arm: did not complete survey n = 2; underage n = 4; attending clinic for TB care n = 49.

From June 2012 to February 2013 we screened 8104 adults upon exit from PHCs of whom 4098 were eligible and offered enrolment (Fig 1). 134 declined enrolment and 3964 were enrolled. A further 360 participants were subsequently withdrawn; one entire cluster where the study protocol was not adhered to (n = 197), participants who were either already on TB treatment or were being investigated for TB prior to the index visit (n = 115), participants enrolled in error either because their age could not be confirmed (n = 30) or were <18 years of age (n = 14) and four participants who did not complete the survey, leaving 3604 participants (1676 in the Xpert arm, 1928 in the microscopy arm) in the analysis.

Among these 3604 participants, the median age was 38 years, and 71.4% were female. Of the 2610 (72.4%) participants willing to share their HIV status 1398 (38.8%) reported being HIV positive and of these, 908 (64.9%) were on antiretroviral therapy (ART) (Table 1). Among all 3604 participants, 70.6% and 30.8% reported cough and fever respectively with similar proportions reporting weight loss and night sweats (42.1% and 41.6% respectively). For 1267 participants (35.2%) the main reason for attending the clinic was due to having at least one TB symptom (Table 1). Overall 2130/3604 (59.1%) had reported TB symptom(s) to HCW. Generally the characteristics were balanced by study arm except that participants in the Xpert arm were less likely to have attended the clinic because of TB symptoms, and, if HIV positive, were less likely to be on (ART).

**Table 1:** Characteristics of participants by arm.

			Xpert	Microscopy
			(n = 1676)	(n = 1928)
Age	in years	median (IQR)	35 (27–48)	41 (30–53)
Sex	Female	% (n)	73.3% (1228)	69.8% (1345)
Country of birth	South Africa	% (n)	93.0% (1558)	95.5% (1842)
Ethnicity	Black	% (n)	98.7% (1654)	96.6% (1863)
Marital status	Single	% (n)	52.3% (877)	55.1% (1063)
Education Level	Grade 0–7	% (n)	34.7% (582)	36.4% (702)
	Grade 8–11	% (n)	42.3% (709)	39.4% (760)
	Grade 12	% (n)	19.9% (334)	20.6% (398)
	Post-secondary	% (n)	3.0% (51)	3.5% (68)
Dwelling	Concrete	% (n)	58.4% (979)	74.7% (1440)
	Traditional	% (n)	27.4% (460)	11.8% (228)
	Informal	% (n)	8.5% (142)	8.5% (164)
	On property/backroom	% (n)	5.7% (95)	5.0% (96)
Symptoms reported				
Cough	Yes	% (n)	68.3% (1144)	72.6% (1399)
Night sweats	Yes	% (n)	39.9% (669)	43.0% (829)
Weight loss	Yes	% (n)	41.1% (688)	43.2% (832)
Fever	Yes	% (n)	22.8% (382)	37.8% (729)
Total number of symptoms^1^	1	% (n)	50.8% (851)	38.9% (749)
	2	% (n)	30.3% (508)	34.1% (658)
	3	% (n)	15.0% (252)	18.7% (360)
	4	% (n)	3.9% (65)	8.4% (161)
Symptoms duration				
Cough	None	% (n)	31.7% (532)	27.4% (528)
	= <2weeks	% (n)	44.4% (744)	50.6% (976)
	>2weeks	% (n)	23.9% (400)	22.0% (424)
Night sweats	None	% (n)	60.1% (1007)	57.0% (1099)
	= <2weeks	% (n)	15.5% (259)	24.3% (468)
	>2weeks	% (n)	24.5% (410)	18.7% (361)
Weight loss	None	% (n)	58.9% (988)	56.9% (1096)
	= <2weeks	% (n)	9.3% (155)	16.3% (315)
	>2weeks	% (n)	31.8% (533)	26.8% (517)
Fever	None	% (n)	77.2% (1294)	62.2% (1199)
	= <2weeks	% (n)	17.1% (286)	27.4% (528)
	>2weeks	% (n)	5.7% (96)	10.4% (201)
Previous tuberculosis	Yes	% (n)	12.7% (213)	17.0% (327)
HIV status, self-report^2^	Negative	%(n)	34.1% (572)	31.7% (611)
	Positive	% (n)	16.4% (275)	11.8% (227)
	Positive on ART	% (n)	22.2% (372)	27.1% (523)
	Unknown	% (n)	27.3% (457)	29.4% (567)
Main reason for visit	TB symptoms	% (n)	31.6% (530)	38.2% (737)
	HIV care	% (n)	21.5% (360)	22.7% (437)
	HIV test	% (n)	5.4% (91)	3.9% (76)
	General/chronic^3^	% (n)	17.8% (298)	19.8% (382)
	ANC	% (n)	5.9% (99)	3.0% (58)
	Other	% (n)	17.8% (298)	12.3% (238)
Reported TB symptoms to health care worker	Yes	% (n)	54.0% (905)	63.5% (1225)

^1^Total number of symptoms based on current cough, night sweats, fever and weight loss; TB = tuberculosis; ART = antiretroviral therapy; ANC = antenatal clinic attendance

^2^ Percentage HIV positive out of those willing to share HIV status 52.6% (647/1232) and 54.5% (749/1375) in the Xpert and microscopy arms, respectively.

^3^Attending clinic for other routine chronic disease follow-up, such as diabetes and hypertension

### Effect of Xpert MTB/RIF on Study Outcomes

Overall 22.7% of participants (818) self-reported being requested to give sputum for TB investigation: 26.0% (436/1676) in Xpert arm compared to 19.8% (382/1928) in the control arm, giving a prevalence ratio [PR] of 1.24 (95% CI 0.65–2.36; p = 0.49). After adjusting for baseline imbalance there was a 31% increase in the percentage of participants having sputum requested in the Xpert compared to control arm (adjusted PR 1.31, 95% CI 0.78–2.20, p = 0.28, Table 2).

**Table 2:** Effect of Xpert on primary and secondary outcomes.

Primary outcome						
	Xpert		Microscopy		Prevalence ratio (95% CI), p-value	
	Sputum requested n/N	%*	Sputum requested n/N	%*	Unadjusted	Adjusted
**Proportion having sputum requested by HCW (n = 3604)**	436/1676	26.0%	382/1928	19.8%	1.24 (0.65–2.36), p = 0.49	1.31 (0.78–2.20), p = 0.28
**Sensitivity analyses**						
**Main reason for attending clinic was TB symptoms (n = 1267)** ^1^	260/530	49.1%	220/737	29.9%	1.45 (0.87–2.40), p = 0.14	1.38 (0.89–2.13), p = 0.14
**Reported TB symptoms to health care worker (n = 2130)** ^2^	384/905	42.4%	345/1225	28.2%	1.56 (1.00–2.43), p = 0.05	1.46 (0.99–2.16), p = 0.05
**Self-reported cough and reported cough to health care workers** ^2^ **(n = 1624)**	338/681	49.6%	299/943	31.7%	1.62 (1.03–2.52), p = 0.04	1.53 (1.02–2.29), p = 0.04
**Living with HIV (n = 1396)** ^2^	190/647	29.4%	156/749	20.8%	1.38 (0.74–2.57), p = 0.29	1.38 (0.88–2.16), p = 0.15

CI = confidence interval

*summary ignores cluster

^1^Adjusted for age group, gender, duration of cough, duration of night sweats, number of WHO TB symptoms, and HIV status with ART

^2^Adjusted for age group, gender, duration of cough, duration of night sweats, number of WHO TB symptoms, reason for attending clinic and HIV status with ART

### Sensitivity Analyses

Restricting to those whose main reason for attending the clinic was because they had TB symptoms (n = 1267), 37.9% had sputum requested by HCW and was higher in Xpert compared to control arm (49.1%, 260/530 vs 29.9%, 220/737), though the CI were wide (adjusted PR 1.38, 95% CI 0.89–2.13, p = 0.14, Table 2).

Among a subgroup who said that they reported TB symptoms to HCWs (n = 2130), 42.4% (384/905) and 28.2% (345/1225) had sputum requested in the Xpert and control arms respectively, giving an adjusted PR of 1.47 (95% CI 0.99–2.16, p = 0.05). Restricting to those who self-reported cough and reported having a cough to health care workers (n = 1624), 49.6% (338/681) and 31.7% (299/943) had sputum requested in the Xpert and control arms respectively, giving an adjusted PR of 1.53 (95% CI 1.01–2.29, p = 0.04).

Of the 1397 participants who reported living with HIV, 29.4% (190/647) vs. 20.8% (156/749) had sputum requested in Xpert and control arms respectively. After adjusting for age, gender, cough duration, duration of night sweats, number of TB symptoms, reason for visiting clinic and whether on ART, the adjusted PR was 1.38 (95% CI 0.88–2.16, p = 0.15, Table 2).

### Factors Associated with Having Sputum Requested by HCW

Among those attending clinic because of TB symptoms (n = 1267), individual level factors associated with higher odds of having sputum requested by HCW included being male (aOR 1.49, 95% CI 1.13–1.97), self-reported HIV positive status and not on ART versus HIV negative (aOR 2.58, 95% CI 1.69–3.92) and if they informed HCW of symptoms (aOR 4.81, 95% CI 2.88–8.03). Participants reporting increasing number of symptoms were more likely to have sputum requested (≥2 vs 1, Table 3). Similarly the longer the duration of cough, night sweats and weight loss (≥2 weeks vs ≤2 weeks) the more likely the participants were to have sputum requested by HCW (Table 3).

**Table 3:** Factors associated with having sputum requested for TB investigations among clients attending clinic because of symptoms suggestive of TB (Xpert and microscopy arms combined, n = 1267).

	Total	Sputum requested by HCW	Unadjusted OR (95% CI)	P value	Adjusted OR* (95% CI)	P value
		n (row %)				
**All participants**	1267	480 (37.9)				
**Age, years**				0.14		0.37
18–29.9	380	139 (36.6)	1		1	
30–34.9	187	71 (38.0)	1.17 (0.79–1.75)		0.93 (0.60–1.43)	
35–39.9	136	61 (44.9)	1.41 (0.91–2.19)		1.17 (0.72–1.89)	
40–49.9	253	108 (42.7)	1.54 (1.07–2.20)		1.23 (0.83–1.81)	
≥50	311	101 (32.5)	1.06 (0.75–1.51)		0.82 (0.57–1.21)	
**Gender**				0.0005		0.0051
Female	813	273 (33.6)	1		1	
Male	454	207 (45.6)	1.58 (1.22- 2.04)		1.49 (1.13–1.97)	
**Cough duration**				<0.0001		<0.0001
No cough	220	41 (18.6)	1		1	
≤2 weeks	656	236 (36.0)	3.04 (2.02–4.58)		3.00 (1.94–4.63)	
>2 weeks	391	203 (51.9)	5.08 (3.30–7.81)		4.63 (2.95–7.28)	
**Duration of night sweats**				<0.0001		0.0001
None	697	216 (31.0)	1		1	
≤2 weeks	299	138 (46.2)	2.05 (1.51–2.78)		1.92 (1.39–2.65)	
>2 weeks	271	126 (46.5)	1.73 (1.25–2.88)		1.62 (1.12–2.34)	
**Weight loss duration**				0.0024		
None	700	229 (32.7)	1			
≤2 weeks	189	86 (45.5)	1.80 (1.26–2.58)			
>2 weeks	378	165 (43.7)	1.39 (1.04–1.86)			
**Number of WHO symptoms**				<0.0001		
One	419	111 (26.5)	1			
Two	430	166 (38.6)	1.96 (1.42–2.69)			
Three	295	137 (46.4)	2.73 (1.92–3.89)			
Four	123	66 (53.7)	3.83 (2.39–6.12)			
**HIV status**				<0.0001		0.0001
HIV negative	458	144 (31.4)	1		1	
HIV +/on ART	167	56 (33.5)	1.27 (0.84–1.91)		1.22 (0.78–1.92)	
HIV +/not on ART	181	111 (61.3)	2.83 (1.91–4.19)		2.58 (1.69–3.92)	
HIV unknown	461	169 (36.7)	1.18 (0.87–1.60)		1.18 (0.86–1.63)	
**Informed health worker of symptoms**				<0.0001		<0.0001
No	188	22 (11.7)	1		1	
Yes	1079	458 (42.5)	5.84 (3.56–9.56)		4.81 (2.88–8.03)	

* controlling for clinic using a random effects model

## Discussion

Our data suggests opportunities are being missed to identify TB cases at PHCs where people present with symptoms suggestive of TB. Our findings show that a large proportion of clinic attendees reporting at least one TB symptom did not have sputum taken for further TB investigations in both the Xpert MTB/RIF and microscopy arm. In this study from a high HIV and TB burden setting where Xpert MTB/RIF assay has been adopted as the initial test for TB, the implementation of Xpert did not change HCW TB investigation practices. Implementation of the new test, Xpert MTB/RIF resulted in a modest increase in the proportion of symptomatic patients having sputum requested for TB investigations. There was some evidence of an effect among those who reported TB symptoms to the HCW with a 47% increase in those having sputum requested in the Xpert arm. Given that Xpert MTB/RIF would have been perceived as an expensive test, HCWs appeared not to have rationed its use at these primary health care clinics.

Having sputum collected for TB investigation among persons with presumed TB is critical step in the continuum of care. Among those who reported a cough to a HCW, less than half had sputum collected for TB tests. This despite South African guidelines for HIV-positive persons [10] and TB infection control advising screening of all persons for TB symptoms at entry to health care facilities [11]. More importantly, these findings suggest failure to request TB tests, even among people reporting a cough to HCWs. Furthermore this also represents poor infection control practice as these potentially infectious persons would ideally have been triaged on entry to minimise transmission to other clinic clients. Other data suggests that active screening may increase the number of cases found and decrease the time to detection [12].

Our data show that one of the strongest factors associated with having sputum sent for TB investigations was if people reported a symptom to the HCW. We did not collect data on why patients did not report symptoms to HCW but it would seem reasonable to suggest that there could be activities at facilities to educate patients about TB symptoms, encourage them to specifically report them and ask for a test for TB. Other studies reporting missed opportunities for diagnosing TB also noted the importance of educating people attending health services about TB symptoms and testing [4]. Interestingly those who reported more symptoms and symptoms of longer duration, were more likely to have sputum requested. As HCWs were not interviewed in this study, we cannot infer why they were more likely to request sputum in such cases but it would seem plausible that the recommendations for TB screening are not being adhered to.

TB screening is recommended for HIV-positive individuals at every clinic visit [13]. In our study HIV-positive individuals were more likely to be investigated for TB if they were not on ART. Screening for TB among HIV-positive individuals is important regardless of whether they are receiving ART or not and also provides an opportunity to evaluate patients for eligibility to start isoniazid preventive therapy.

Our study has limitations; we enrolled people exiting PHCs and depended on self-report by the participants to determine whether or not a sputum sample was requested. This information was not verified by clinic record review. Among those screened for eligibility to the study, the proportion of participants reporting TB symptoms (50%) seems very high and we could not verify if study staff at times pre-screened participants. Study staff were trained continuously during the study and monitoring visits conducted.

The proportion having sputum requested was low in both Xpert and microscopy arms even among those whose main reason for attending clinic was because of TB symptoms. In settings of high TB prevalence, undetected TB is frequently identified when intensified screening is done in household contacts [14,15] and newly diagnosed HIV-positive individuals attending HIV testing and counselling [16, 17,18]. Not investigating people presenting at clinics with symptoms suggestive of TB presents a missed opportunity for diagnosing TB. In South Africa where HIV is also prevalent, with services integrated at primary health care level, investigating clinic attendees for TB also provides an opportunity for raising awareness of TB symptoms and linkage to HIV testing and care services.

## Conclusions

In conclusion many people who have at least one TB symptom are not being tested at PHCs, representing a missed opportunity for TB diagnosis. However from the study we cannot determine who actually had TB and have no way of knowing what would have happened if every symptomatic person had been tested for TB. Testing everyone reporting a TB symptom would be expensive especially with Xpert MTB/RIF and might not be cost effective. There is an urgent need to identify who should be prioritised for testing among people presenting at PHC with symptoms suggestive of TB. Reporting TB symptoms to health care workers was an important determinant for having sputum collected for TB investigations supporting the notion that educating patients and empowering them to better report symptoms when they attend clinics could improve investigation practices. However this needs to be done alongside better adherence to investigation algorithms and emphasising to HCW that we must diagnose and treat TB as early as possible.

## Supporting Information

S1 Table Minimal dataset for variables reported in exit interview substudy. (CSV)

S1 Text Data dictionary—Variable names, description and value labels for exit interview substudy. (TXT)
